# Acute hypoxia modulate macrophage phenotype accompanied with transcriptome re-programming and metabolic re-modeling

**DOI:** 10.3389/fimmu.2025.1534009

**Published:** 2025-02-17

**Authors:** Binda Sun, Yao Long, Gang Xu, Jian Chen, Gang Wu, Bao Liu, Yuqi Gao

**Affiliations:** ^1^ Institute of Medicine and Equipment for High Altitude Region, College of High Altitude Military Medicine, Army Medical University (Third Military Medical University), Chongqing, China; ^2^ Key Laboratory of Extreme Environmental Medicine, Ministry of Education of China, Chongqing, China; ^3^ Key Laboratory of High Altitude Medicine, Chinese People’s Liberation Army (PLA), Chongqing, China; ^4^ Department of High Altitude Physiology and Pathology, College of High Altitude Military Medicine, Army Medical University (Third Military Medical University), Chongqing, China

**Keywords:** acute hypoxia, macrophage, transcriptome reprogramming, metabolic remodeling, pentose phosphate pathway

## Abstract

**Introduction:**

Macrophages, which tend to aggregate in the hypoxic regions of tissues, have a significant impact on disease progression and outcome because of their plastic responsiveness to hypoxia, particularly in the early stages. Understanding macrophages’participation in hypoxia-related disorders requires demonstrating the impact of acute hypoxia on their survival, phenotype, and function.

**Methods:**

Here we conducted a systematic evaluation of macrophage responses to hypoxia over 24 and 48 h including cell growth and activity, inflamatory response, macrophage polarization and transcriptional and metabolic changes.

**Results:**

We found that acute hypoxia suppresses macrophage proliferation and phagocytosis function with a parallel change of transcriptome re-programming and metabolic re-modeling. Although macrophages accumulate transcriptome heterogeneity based on oxygen concentration and culture period, genes involved in hypoxia response, chemotaxis, and glycolytic process were commonly altered during acute hypoxia. Furthermore, the pro-inflammatory response of macrophages was activated during acute hypoxia concomitantly with an enhanced anti-inflammatory regulatory mechanism characterized by increased M2 macrophage population and anti-inflammatory metabolite itaconic acid. Aside from increased glycolysis, the key intermediates in the pentose phosphate pathway significantly increased, such as fructose 1,6-bisphosphate (fold change: 7.8), 6-phosphogluconate (fold change: 6.1), and ribose 5-phosphate (fold change: 3.9), which indicated that the pentose phosphate pathway was an important compensatory metabolic regulation that rules for the response of macrophages to acute hypoxia.

**Discussion:**

These findings highlight that acute hypoxia suppresses macrophage viability and phagocytosis, while acute hypoxia modifies the transcriptome and metabolome in specific inflammatory responses and metabolic pathways to facilitate the adaptation of macrophage in hypoxic conditions.

## Introduction

Macrophages, originating from the hematopoietic system, are a pillar of both the innate and adaptive immune responses, contributing to essential physiologic functions such as immune defense against internal and external environmental stressors, tissue homeostasis, and repair (1). These cells are prominently found in hypoxic regions associated with various pathological conditions, including various solid tumors, atherosclerotic plaques, myocardial infarction, wound healing, and arthritic joints (2–7). The presence of macrophages in these hypoxic niches underscores their involvement in the progression and prognosis of these diseases. The notion was further confirmed by recent single-cell RNA sequencing assays which have also delineated the high plasticity of macrophages with a far more complex and specific landscape of interconnected molecules (2, 8–10). Given the tendency of macrophages to accumulate in oxygen-deprived environments, macrophages have been proposed as potential delivery vehicles for hypoxia-regulated gene therapy, particularly in the early stages of the disease.

Macrophages exhibit remarkable phenotypic and functional diversity due to their ability to adapt to the micro-environmental factors that they encounter. Hypoxia, characterized by an imbalance between oxygen supply and demand, is a critical physiological or pathological stressor that profoundly influences cellular activities and systemic responses across various organisms (11, 12). A substantial body of literature has demonstrated that the hypoxia-inducible factors (HIF) family of transcription factors plays a critical regulatory role in how macrophages respond to hypoxia (13–15). HIFs serve as the primary mediators of cellular adaptation to severe oxygen deprivation by regulating a range of target genes involved in glycolysis and angiogenesis (16). They have also been identified as essential transcriptional factors that influence macrophage viability, inflammatory responses, and phagocytic activity (13, 17). However, with the application of omics technologies, new hypoxia-regulated molecules are continuously being discovered. A recent transcriptomic analyses of human blood under acute systemic hypoxia, such as that experienced at high altitudes, revealed that the transcriptional response is largely independent of HIF1A (18). The different duration of hypoxic exposure elicits distinct transcriptional responses in macrophages, reflecting varying energy demands and functional requirements (19–21), and Hiroki Sekine et al. identified that the PNPO–PLP axis is independent of the HIF pathway, which senses prolonged hypoxia in macrophages (20). These studies not only indicate the existence of non-HIF regulatory mechanisms in the hypoxic response of macrophages but also suggest that hypoxia-induced alterations in macrophages are complicated and diverse, with close ties to oxygen concentration and exposure period. To fully understand how macrophages influence the initiation and progression of diseases, it is crucial to elucidate their early responses to hypoxia, a process that remains incompletely characterized.

In this study, we differentiated macrophages from mouse bone marrow and cultured them at 1% oxygen concentration, which is the physiological level of hypoxia found in many tissues and malignancies, and oxidative phosphorylation still proceeds under this condition (22, 23). Our findings reveal that acute hypoxia significantly impacts macrophage survival, energy metabolism, inflammatory responses, and phagocytic function. Using RNA sequencing, we analyzed transcriptome-wide changes in primary mouse macrophages exposed to 1% and 3% oxygen for 24 and 48 h. The results demonstrate that the transcriptional response of macrophages to acute hypoxia is highly dependent on both oxygen concentration and exposure duration, with oxygen concentration having a greater impact than exposure time. Furthermore, targeted metabolic assays confirm that a shift toward glycolysis and pentose phosphate pathway is a hallmark of macrophage adaptation to acute hypoxia.

## Materials and methods

### Animals

C57BL/6N mice (SPF grade, male, 6–8 weeks old) were purchased from Changzhou Kavens Laboratory Animal Company and maintained in a temperature- and humidity-controlled room with a 12-h light–dark cycle. All animal procedures were approved by the Committee of Third Military Medical University.

### Cell lines

RAW264.7 (mouse macrophages) and L-929 (mouse fibroblasts) were obtained from ATCC and handled according to the instructions provided on the ATCC product sheet.

### Bone-marrow isolation and differentiation to bone marrow-derived macrophages

Bone marrow (BM) cells from 8- to 10-week-old mice were isolated by flushing the femurs and tibiae with DMEM basal medium using 10-mL syringes with 22 G needles (day 0) and subjected to red blood cell lysis with ACK lysis buffer prior to neutralization and resuspension in DMEM basal medium. The isolated BM cells were counted using a hemocytometer and cultured at 37°C, 5% CO_2_ in 10-cm tissue culture plates at a density of 3 million cells/plate for 7 days. The cells were cultured with 10 ng/mL m-CSF (576404, Biolegend) for the first 3 days, followed by 30% (v/v) L-929 fibroblast medium supernatant for the next 4 days to induce differentiation into BMDMs. During differentiation, DMEM was supplemented with 10% (v/v) heat-inactivated fetal bovine serum. *In vitro* assays were performed on BMDMs from day 7 to day 9. Macrophage differentiation was confirmed by observing adherent cells under an inverted microscope (Olympus CKX41, Japan). Additionally, macrophage-specific markers (F4/80 and CD11b) were identified using flow cytometry.

### Hypoxia exposure

On day 7 of culture, BMDM cells were placed in a hypoxia workstation (Ruskinn InvivO2 1000, English). Petri dishes containing cultured cells were placed in the chamber. Media and trypsin were equilibrated to the atmosphere in the workstation before being added to the cells.

### Cell growth assay

For the 5-ethynyl-2′-deoxyuridine (EdU) incorporation assay, the proliferating cells were evaluated using the BeyoClick™ EdU Cell Proliferation Kit with Alexa Fluor 594 (C0078S, Beyotime). Briefly, after incubation with 10 µM EdU for 24 h under hypoxia or normoxia conditions, the BMDM cells were fixed with 4% paraformaldehyde, permeabilized with 0.3% Triton X-100, and stained with fluorescent dyes. Hoechst 33342 was used to stain the cell nuclei for 10 min at room temperature. Subsequently, the cells were observed under a fluorescent microscope (Leica DMI8, Germany) in 15 random fields. The cell proliferation rate was calculated with the ImageJ software as the percentage of EdU-positive cells relative to the total number of cells.

### Reactive oxygen species measurement

Cells are collected and tested using Reactive Oxygen Species Assay Kit (S0033S, Beyotime) following the manufacturer’s instructions.

### Lactate dehydrogenase analysis

The concentration of lactate dehydrogenase (LDH) in the culture medium was determined using Lactate Dehydrogenase Assay Kit (A020-2-2, Jiancheng Bioengineering). The assay was conducted in strict accordance with the manufacturer’s instructions.

### Ratio of NAD+/NADH assay

Cultured BMDM cells were collected after the respective treatments. The NAD+/NADH ratio was measured using NAD+/NADH Assay Kit with WST-8 (S0175, Beyotime) according to the manufacturer’s directions.

### Phagocytosis assay

The macrophage phagocytic activity of bone-marrow-derived macrophages (BMDMs) was tested using the Vybrant Phagocytosis Assay kit (Thermo Fisher Scientific, v-6694) following the manufacturer’s instructions. Briefly, BMDMs were harvested after exposure to normoxia and hypoxia for 24 or 48 h, respectively, and seeded in 96-well white culture plates at 105 cells per well for 2 h. The culture medium was then removed, and 100 μL of fluorescein-labeled *E. coli* BioParticle suspension was added to each well, followed by incubation under normoxia or hypoxia conditions. After 2 h, the supernatant was removed by vacuum aspiration, and 100 μL of the prepared trypan blue suspension was immediately added to all wells for 1 min. Excess trypan blue dye was removed by aspiration, and the plates were read on a GloMAX Discover microplate reader (Promega, USA) with excitation at 480 nm and emission at 520 nm.

### Cell cycle analyses

The cell cycle was assessed using Cell Cycle Test Kit (DW0108, Dowobio) following the manufacturer’s instructions. Briefly, BMDMs were trypsinized and resuspended in 0.5 mL of cold PBS after exposure to normoxia and hypoxia for 24 or 48 h, respectively. Subsequently, the cells were fixed with 1 mL of ice-cold 80% ethanol and incubated overnight at 4°C. The cells were then washed twice with ice-cold PBS and resuspended in 0.5 mL of ice-cold PBS. Subsequently, 20 μL of RNaseA was added to the samples and incubated at 37°C for 30 min. Following this, 500 μL of propidium iodide was added and incubated at 4°C for 2 h. Finally, the cells were resuspended in 500 μL of ice-cold PBS and analyzed by flow cytometry.

### Protein extraction and western blotting

Whole-cell lysates obtained by cell pellets were homogenized in RIPA buffer (P0013B, Beyotime) with a freshly added protease inhibitor cocktail (HY-K0010, MCE), phosphatase inhibitor cocktail I (MCE, HY-K0021), and phosphatase inhibitor cocktail II (HY-K0022, MCE). Western blotting analyses were performed with anti-IL-1β (12242s, Cell Signaling Technology), anti-IL10 (82191-3-RR, Proteintech), anti- TGF-β1 (81746-2-RR, Proteintech), anti-HSP90 (A5027, Abclonal), and anti-tubulin (A6830, Abclonal).

### RNA extraction and real-time PCR for mRNA

RNA was extracted from RAW264.7 and BMDMs using the universal RNA extraction reagent TRIzol (15596018, Invitrogen). Subsequently, a reverse transcriptase reaction was performed following the manufacturer’s protocol with the PrimeScriptTM FAST RT reagent kit with gDNA Eraser (RR092A, Takara). The resulting cDNA was then combined with TB Green Premix Ex TaqTM II (RR820A, Takara), as per the manufacturer’s instructions, along with 4 pmol of both forward and reverse primers to quantify the mRNA levels of mouse genes. All primers utilized in real-time reactions were procured from Invitrogen; their names and sequences can be found in **Supplementary Table S1**. qPCR was conducted on CFX Connect Real-Time System (BIO-RAD, USA) with the amplification steps set at 95°C for 2 min, followed by 40 cycles of 9°C for 5 s and 60°C for 30 s. The relative quantification of mRNA expression levels was determined using the comparative threshold cycle method with β-actin for normalization.

### Transwell migration assay

Macrophage migration was assessed using a 24-well Transwell insert (8-µm pores, 353097, Corning) following exposure to normoxic or hypoxic conditions for 24 or 48 h. Briefly, bone-marrow-derived macrophages (BMDMs) were harvested after exposure to normoxia or hypoxia for 24 or 48 h. A total of 2 × 10^5^ macrophages in 200 µL of DMEM containing 1% antibiotics were seeded into the upper chamber. The lower chamber was filled with 800 µL of DMEM medium containing 20% fetal bovine serum (FBS), 1% antibiotics, and 40 ng/mL M-CSF. After 12 h of migration, non-migrated cells in the upper chamber were removed using a cotton swab. The lower surface of the Transwell membrane was then fixed with 4% paraformaldehyde (PFA) for 30 min and stained with crystal violet (C0121, Beyotime) for 20 min. Five images of randomly selected fields were captured under a light microscope for subsequent quantification.

### Flow cytometry analysis

Macrophage polarization was evaluated by flow cytometry. Briefly, the samples were blocked with anti-mouse CD16/32 (101320, Biolegend) to reduce nonspecific antibody binding for 15 min at 4°C. The cells were then incubated with fluorochrome-conjugated antibodies for 30 min at 4°C. The antibodies used included anti-mouse F4/80 Brilliant Violet 421 (123132, Biolegend), anti-mouse CD11b Alexa Fluor™ 700 (2433037, Invitrogen), APC anti-mouse CD206 (123132, Biolegend), and Brilliant Violet 650 anti-mouse CD86 (105035, Biolegend). Flow cytometry was performed using a Fongcyte™ S flow cytometer (Challenbio, Beijing), and data were analyzed using the manufacturer-provided ModelFlower software.

### Enzyme-linked immunosorbent assay

IL10, TGF-β1, IL-6, and IL-1β concentrations in BMDM culture medium were measured using IL10, TGF-β1, IL-6, and IL-1β mouse-uncoated ELISA Kit (BMS614, Invitrogen, EK0515/EK0411/EK0394, Boster) according to the manufacturer’s instructions. The absorbance values were measured at a wavelength of 450 nm using a microplate reader (Thermo, USA).

### Transcriptome sequencing (RNA-seq) and analysis

RNA-seq analysis was done on BMDMs. BMDMs were cultured at different oxygen concentrations (1% O_2_ and 3% O_2_) for 24 and 48 h. Total RNA was isolated using TRIzol (Invitrogen, Carlsbad, CA, USA) according to the instructions in the manual. Total RNA was qualified and quantified using a Nano Drop and Agilent 2100 bioanalyzer (Thermo Fisher Scientific, MA, USA). Oligo(dT)-attached magnetic beads were used to purify mRNA from total RNA to prepare a RNA-seq library with the following steps: including mRNA fragment, cDNA synthesis, end repair, add A and adaptor ligation, PCR and purified its product with Ampure XP Beads (AGENCOURT), library quality validated on the Agilent Technologies 2100 bioanalyzer, and circularization to format the final library. The libraries were amplified with phi29 to make a DNA nanoball (DNB) which had more than 300 copies of one molecular. DNBs were loaded into the patterned nanoarray, and pair-end 150 bases reads were generated on MGISEQ-2000 platform (BGI-Shenzhen, China).

Raw fastq reads were filtered by using SOAPnuke to remove low-quality base pairs with the following parameters: -l 15 –q 0.2 –n 0.05. This made sure that all of the reads had no sequencing adapter, and reads with low-quality base ratio more than 20% and unknown base (“N” base) ratio more than 5% will be removed. Then, the reads were mapped to the mouse genome mm10 using HISAT2 to identify alternative splicing sites using the following parameters: –sensitive –no-discordant –no-mixed –l 1 –X 1000 –p 8 –rna-strandness RF. Bowtie2 was applied to align the reads to the gene set with parameters as –q –phred64 –sensitive –dpad 0 –gbar 99999999 –map 1,1 –np 1 –X 1000 –no-mixed –no-discordant –p 1 –k 200. The aligned bam files were then sorted using samtools. The expression level of the gene was calculated by RSEM (v1.3.1). Essentially, differential expression analysis was done using the R statistical package DESeq2 (v1.4.5), and differentially expressed genes were called if they had |fold change| ι1 and FDR <0.05. Gene Ontology (GO) (http://www.geneontology.org) and Kyoto Encyclopedia of Genes and Genomes (KEGG) (https://www.kegg.jp/) enrichment analysis of annotated differentially expressed genes was performed using the clusterProfiler package (v4.12.0) based on the hypergeometric test to gain insight into the changes in phenotype. Enrichment of DEGs was performed in Enrichr against the ENCODE and ChEA consensus transcription factor gene set library.

### Fuzzy C-means clustering

Genes from various oxygen concentrations and exposure durations were categorized into distinct clusters using the Mfuzz package in R with the fuzzy c-means algorithm.

### Metabolite extraction and LC–MS

The cells were counted, and their viability was calculated. The cell count was multiplied by the peak volume (size), and this product was used to calculate the volume of solvent containing methanol, acetonitrile, and water (2:2:1, v/v) for extraction. For absolute quantification of the metabolites, stock solutions of stable-isotope internal standards were added to the extraction solvent simultaneously. Then, the samples were subjected to vigorous shaking for 2 min at 4°C, incubated on ice for 20 min, and then centrifuged at 14,000 *g* for 20 min at 4°C. The supernatant was collected and flowed through a 96-well protein precipitation plate, and then the elution was collected and dried in a vacuum centrifuge at 4°C. The samples were re-dissolved in 100 μL acetonitrile/water (1:1, v/v) solvent and centrifuged at 14,000 *g* at 4°C for 15 min for LC–MS analysis, and then the supernatant was injected.

LC–MS analyses were done using an UHPLC (1290 Infinity LC, Agilent Technologies) coupled to a QTRAP MS (6500+, Sciex) in Shanghai Applied Protein Technology Co., Ltd. The analytes were separated on HILIC (Waters UPLC BEH Amide column, 2.1 mm × 100 mm, 1.7 μm) and C18 columns (Waters UPLC BEH C18-2.1 mm × 100 mm, 1.7 μm). For HILIC separation, the column temperature was set at 35°C, and the injection volume was 2 μL (mobile phase A: 90% H_2_O + 2 mM ammonium formate + 10% acetonitrile; mobile phase B: 0.4% formic acid in acetonitrile). A gradient (85% B at 0-1 min, 80% B at 3 to 4 min, 70% B at 6 min, 50% B at 10–15.5 min, and 85% B at 15.6–23 min) was then initiated at a flow rate of 300 μL/min. For RPLC separation, the column temperature was set at 40°C, and the injection volume was 2 μL (mobile phase A: 5 mM ammonium acetate in water; mobile phase B: 99.5% acetonitrile). A gradient (5% B at 0 min, 60% B at 5 min, 100% B at 11–13 min, and 5% B at 13.1–13 min) was then initiated at a flow rate of 400 μL/min. The sample was placed at 4°C during the whole analysis process. Moreover, 6500+ QTRAP (AB SCIEX) was performed in positive and negative switch mode. The ESI positive source conditions were as follows: source temperature, 580°C; ion source gas 1 (GS1), 45; ion source gas 2 (GS2), 60; curtain gas (CUR), 35; and ion spray voltage (IS), +4,500 V. The ESI negative source conditions were as follows: source temperature, 580°C; ion source gas 1 (GS1), 45; ion source gas 2 (GS2), 60; curtain gas (CUR), 35; and ion spray voltage (IS), -4,500 V. MRM method was used for mass spectrometry quantitative data acquisition. Polled quality control (QC) samples were set in the sample queue to evaluate the stability and repeatability of the system. MultiQuant and Analyst were used for quantitative data processing.

### Statistical analysis

Statistical analyses were performed with Prism version 9 and R software (v4.4.0). Data were presented graphically as mean ± SEM. The metabolite data was uploaded before importing into SIMCA-P (v14.1, Umetrics, Umea, Sweden) after sum-normalization, where it was subjected to multivariate data analysis, including Pareto-scaled principal component analysis and orthogonal partial least-squares discriminant analysis. The sevenfold cross-validation and response permutation testing were used to evaluate the robustness of the model. The statistical significance of differences between indicated samples was determined by unpaired Student’s *t*-test or one-way ANOVA. *P*-value <0.05 was considered significant.

## Results

### Acute hypoxia inhibited macrophage cell growth and activity

**Figure 1:**
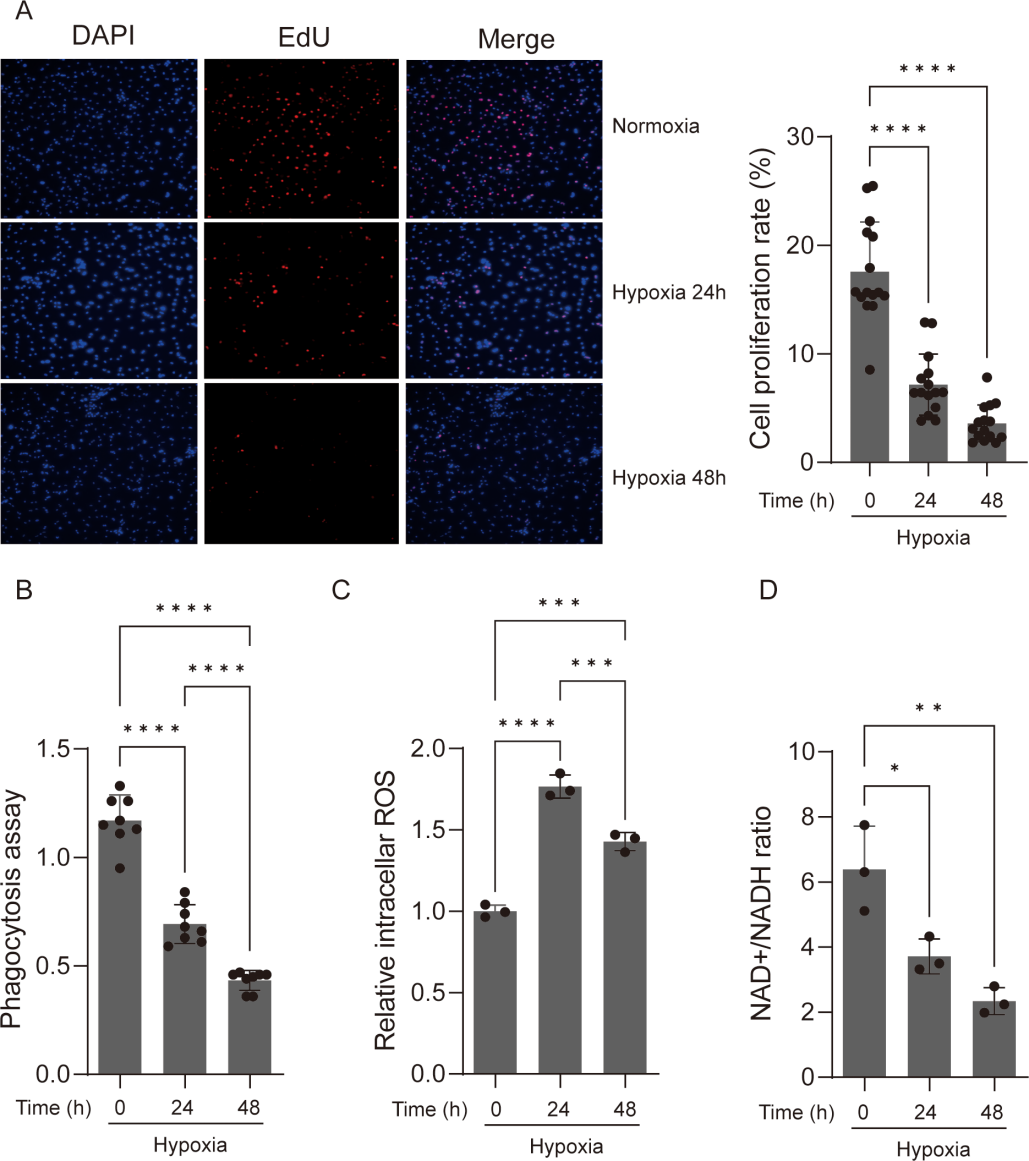
Acute hypoxia suppresses the viability and function of macrophage. **(A)** EdU assay was used to determine the cell proliferation of macrophages. The proliferative cell nuclei were stained using the EdU assay, with a red stain for proliferative cell nuclei and a blue stain for all nuclei using DAPI (original magnification, ×100). The cell proliferation rate was then calculated. Data are from three independent experiments, shown as mean ± SEM. **(B)** Macrophages under normoxic and hypoxic conditions were assayed for phagocytosis of fluorescent *Escherichia coli* particles over a 24-h period. Data are from three independent experiments, shown as mean ± SEM. **(C)** ROS production levels were detected in macrophages under normoxic and hypoxic conditions. Data are from three independent experiments, shown as mean ± SEM. **(D)** NAD+/NADH ratio of macrophages under normoxic and hypoxic conditions. Data are from three independent experiments, shown as mean ± SEM. Significance was determined by one-way ANOVA in **(A–D)**. *p < 0.05, **p < 0.01, ***p < 0.001, ****p < 0.0001.

To investigate the biological behavior of macrophages under acute hypoxia, Edu cell proliferation assay was used to investigate the proliferation of the primary mice macrophages and RAW 264.7 cells that were deployed under 1% oxygen for 24 and 48 h, and the results showed that the growth rate of primary mice macrophages and RAW264.7 cells was significantly reduced by nearly half after hypoxia exposure for both 24 and 48 h (**Figure 1A**; **Supplementary Figure S1A**). Phagocytosis is a crucial macrophage activity that contributes to both health preservation and disease pathogenesis (24). We examined the ability of hypoxic and normoxic macrophages to engulf fluorescent *E. coil* particles following hypoxia incubation. Hypoxic BMDM cells displayed a considerable drop in phagocytic activity after 24 and 48 h of hypoxia exposure (**Figure 1B**), although this was not observed in RAW264.7 cells (**Supplementary Figure S1B**). These findings showed that acute hypoxia has a different effect on macrophage biological behavior in primary cells and cell lines. Acute hypoxia decreased BMDM cell proliferation; moreover, macrophage phagocytosis of microorganisms was lost in BMDM that survived hypoxia incubation.

When the availability of oxygen, the last electron acceptor in mitochondrial energy production, is insufficient, intracellular reactive oxygen species (ROS) production increases, causing oxidative stress and cell damage (25, 26). The increased formation of cellular ROS is linked to a decrease in nicotinamide adenine dinucleotide (NAD^+^) levels (27). The depletion of NAD^+^ will impair macrophage phagocytosis and the resolution of inflammation (28). To determine whether changes in ROS and NAD^+^ are accompanied with the decrease in macrophage proliferation, we compared the ROS and NAD^+^ levels of hypoxic and normoxic macrophages. As expected, the ROS levels significantly increased after hypoxic incubation, while the NAD^+^ levels significantly decreased in BMDM cells (**Figures 1C, D**). However, we observed significantly increased ROS and NAD^+^ levels in RAW264.7 cells after hypoxic incubation, which is consistent with the phagocytic activity observed (**Supplementary Figures S1C, D**).

### Acute-hypoxia-derived inflammatory response of macrophages

**Figure 2:**
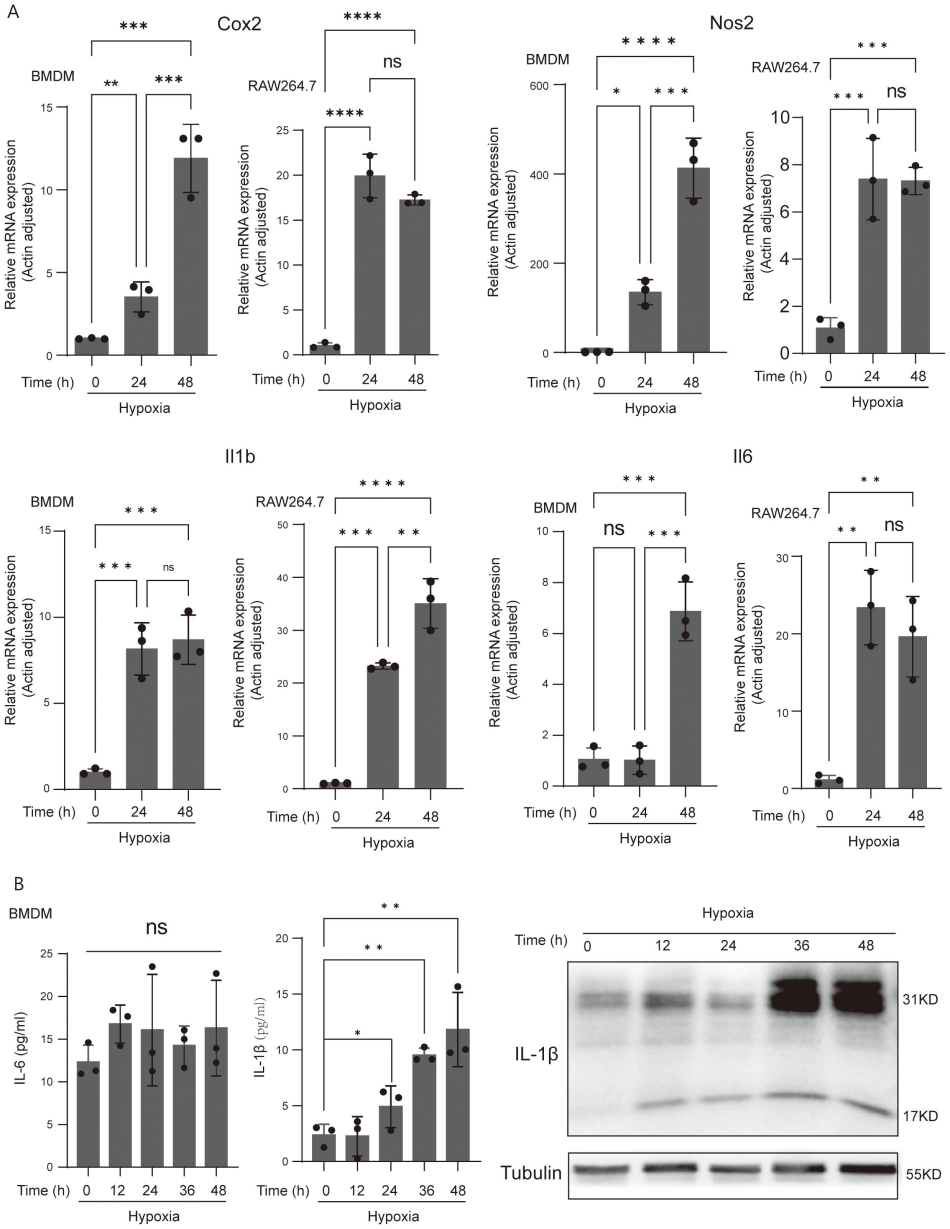
Acute-hypoxia-derived macrophage activation and induced IL-1β gene expression and secretion. **(A)** qPCR analysis of Cox2, Nos2, IL-6, and IL-1β (pro-inflammatory macrophage markers) mRNA levels in resting and hypoxia-activated RAW264.7 cells and BMDMs cultured for 24 and 48 h. Data are from three independent experiments, shown as mean ± SEM. **(B)** The protein abundance of IL-6 and IL-1β of BMDMs. Data are from three independent experiments, shown as mean ± SEM. Significance was determined by one-way ANOVA in **(A, B)**. *p < 0.05, **p < 0.01, ***p < 0.001, ****p < 0.0001, ns, no signficance.

Both innate and adaptive immune responses depend heavily on macrophages. We measured the expression of the pro-inflammatory marker genes, Cox2, Nos2, Il6, and Il1b, in BMDM cells and RAW264.7 cells to determine whether acute hypoxia has an effective effect on the pro-inflammatory phenotype of macrophages. We found that after 24 and 48 h of incubation with 1% oxygen, the expression of Cox2, Nos2, Il6, and Il1b was significantly increased in both BMDM cells and RAW264.7 cells, with the exception of Il6, which showed no changes in BMDM cells following a 24-h hypoxia exposure (**Figure 2A**). Over the course of 48 h, we also measured the protein level expression of pro-inflammatory cytokines, Il6 and Il1b, in BMDM cells every 12 h. The results indicated that whereas IL6 secretion did not considerably change during the hypoxic incubation period, IL-1β secretion increased significantly after 24 h of hypoxia culture, with robust synthesis starting to increase significantly at 12 h (**Figure 2B**). Meanwhile, we observed a significant increase in LDH activity in macrophages cultured under 1% oxygen, with the most pronounced effects at 24 and 48 h (**Supplementary Figure S2**). This may suggests enhanced membrane permeability and aggravated cellular damage under acute hypoxic conditions, which may further influence the synthesis and secretion of IL-1β.

### Reprogramming of the macrophage transcriptome brought on by acute hypoxia

**Figure 3:**
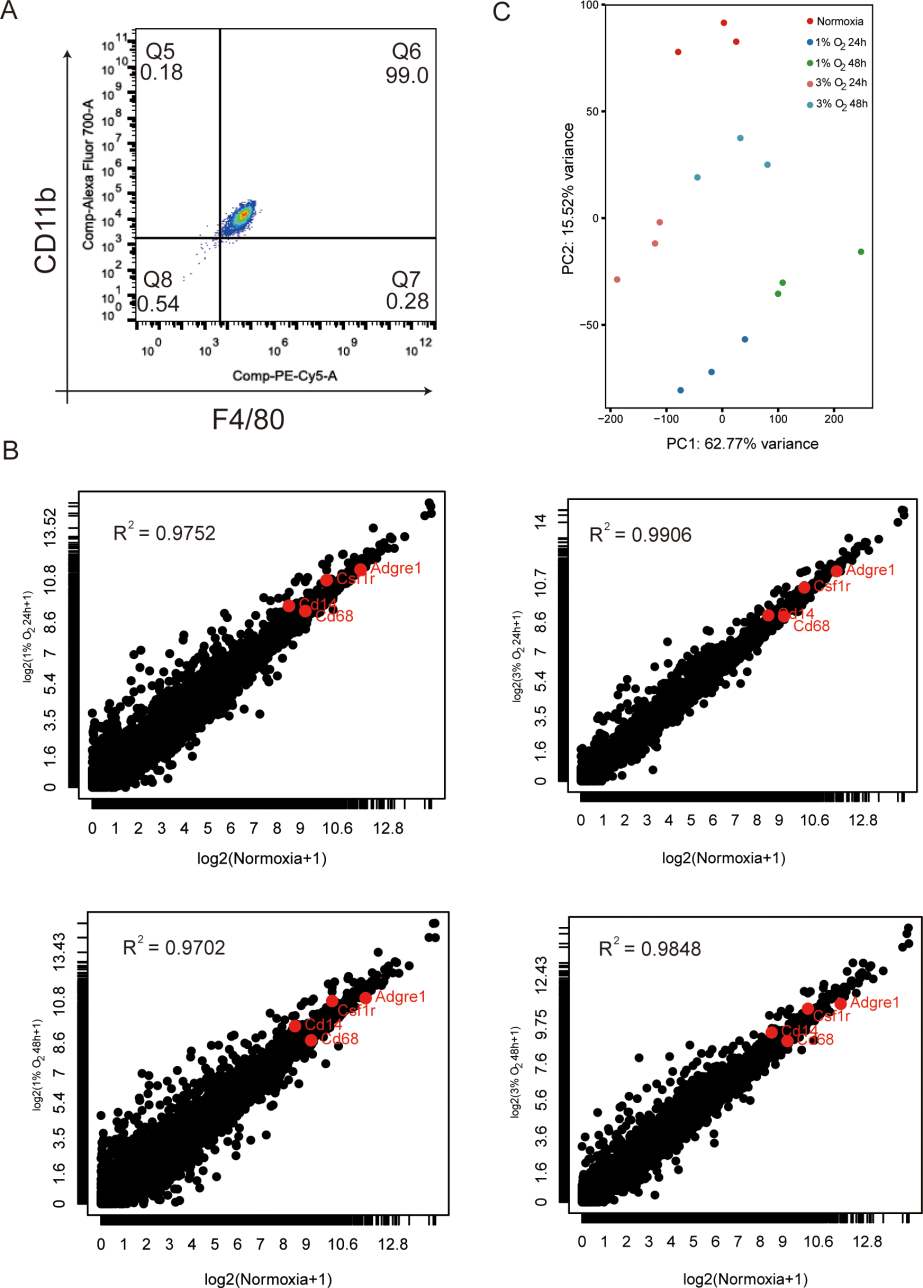
Characterization of BMDM transcriptome at normoxia and after exposure to various oxygen concentrations cultured for 24 and 48 h. **(A)** Representative FACS plots reveal that more than 90% of BMDMs are F4/80+CD11b+ by day 6 of culture. **(B)** Normalized results of RNA-seq (TPM) of total mRNA pools for macrophages under normoxic and various hypoxia conditions. The data are mean of three independently processed samples. Red-enriched dots indicate the abundance of mRNA-encoding macrophage markers Adgre1 (F4/80), Csf1r (c-fms or colony stimulating factor 1 receptor), CD14, and CD68. **(C)** BMDMs from different oxygen concentrations cultured for different times and analyzed by principal component analysis. The effect of oxygen concentration on BMDM gene expression is greater than that of hypoxia exposure time.

The appearance and behavior of a cell are dictated largely by its genetic information flows sourced from genes. We cultivated macrophages from the adult bone marrow of mice in the presence of conditioned medium from L929 fibroblasts with M-CSF for 7 days to assess mRNA transcription in hypoxic vs. normoxic macrophages. The well-known indicators for differentiated macrophages, CD11b and F4/80, were positively expressed by over 90% of the cells in the culture (**Figure 3A**). Using a hypoxia workstation, we exposed differentiated BMDM cells to 1% and 3% oxygen for 24 and 48 h. RNA-seq analyses was applied to assess the genetic information flow from DNA to RNA with the extracted total RNA pools. Additionally, transcripts per kilobase million (TPM) was used to calculate the abundance of each mapped transcript. The total RNA pools for macrophages under normoxic and hypoxic circumstances showed substantial abundances of the mRNAs encoding macrophage-specific markers Adgre1 (F4/80), Csf1r, CD14, and CD68 (**Figure 3B**). Principal components analysis (PCA) results based on whole-transcriptome gene expression levels revealed that normoxic BMDM cells clustered far from hypoxic BMDM cells and that BMDM cells cultured in 1% oxygen were farther away from BMDM cells cultured in a normoxic condition than the BMDM cells cultured in 3% oxygen. There are no discernible differences between BMDM cells cultured at the same oxygen concentration at different times (**Figure 3C**). These findings showed that acute hypoxia caused reprogramming in the transcriptome of macrophages and that oxygen concentration had a stronger impact on the transcriptome of macrophages than exposure duration to hypoxia.

**Figure 4:**
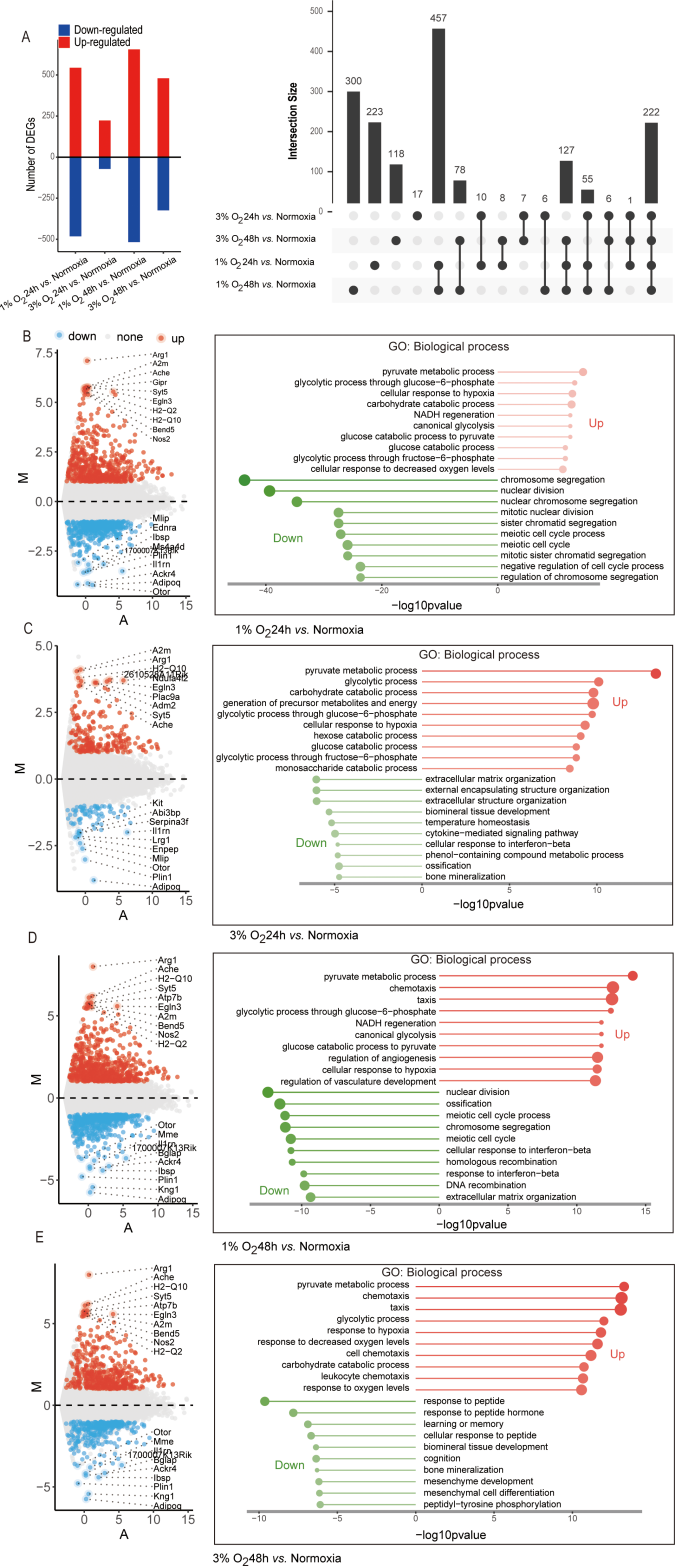
Diagram of differentially expressed genes of the four differential analysis methods (1% O_2_ 24 h vs. normoxia, 1% O_2_ 48 h vs. normoxia, 3% O_2_ 24 h vs. normoxia, and 3% O_2_ 48 h vs. normoxia) and Gene Ontology (biological process) analysis of upregulated and downregulated genes. **(A)** Number of upregulated and downregulated genes in each group. The differentially expressed genes in the four comparisons are shown in the upset plot. The black bars above represent the number of common genes. **(B–E)** Gene Ontology (biological process) analysis of upregulated and downregulated genes and the top 10 upregulated and downregulated differentially expressed genes with the greatest fold change in each group displayed in MA plot. The x-axis and the y-axis represent the average normalized read number and log-intensity ratios, respectively.

We used DESeq2, a commonly used method for RNA-seq differential expression analysis that includes hypoxic differential expression data (21) to examine our data for differentially expressed genes (DEG) in order to learn more about the transcriptional alterations of macrophages during acute hypoxia. The 1% oxygen incubation for 48 h produced the highest DEG (total: 1,173, up: 656, down: 517) when compared to the normoxia group. This was followed by the 1% oxygen incubation for 24 h (total: 1,026, up: 544, down: 482), the 3% oxygen incubation for 48 h (total: 804, up: 480, down: 324), and the 3% oxygen incubation for 24 h (total: 295, up: 223, down: 72) based on the criteria of the absolute value of log2 (fold change [FC]) equal or greater than 1 and false discovery rate [FDR] <0.05 (**Figure 4A**). Quantitative PCR with reverse transcription (RT-qPCR) was used to independently and randomly confirm the oxygen concentration–exposure time specificity of the chosen DEGs (**Supplementary Figures S3A, B**). The right panel of **Figure 4A** displays an UpSet plot showing the number of DEGs shared among the four datasets. The four datasets that were determined to be functionally enriched in response to hypoxia, chemotaxis, and glycolytic process coincided with 222 (13.6%, 222/1,635) DEGs (**Figure 4A**; **Supplementary Figure S4A**). In addition, we found that distinct DEGs correspond to varying oxygen concentrations and durations of hypoxia exposure. DEGs (*n* = 300) specific to 1% oxygen exposure for 48 h were found to be enriched for the regulation of inflammatory response and defense response to the virus, while DEGs (*n* = 118) specific to 3% oxygen exposure for 48 h were functionally enriched in ROS metabolic process and chemotaxis. DEGs (*n* = 223) specific to 1% oxygen exposure for 24 h were enriched in terms of cell cycle and chromosome segregation according to a Gene Ontology (GO) biological process enrichment analysis (**Figure 4A**; **Supplementary Figure S4A**). There are only 17 DEGs that were not functionally enriched in any biological processes and were specific to 3% oxygen exposure for 24 h. Furthermore, we observed that almost one-third of genes (28%, 457/1,635) that were enriched in GO keywords characterizing the mitotic cell cycle were only differently expressed in 1% oxygen at 24 and 48 h, not in 3% oxygen (**Figure 4A**; **Supplementary Figure S4A**). The cell’s transcriptional program in response to signaling events was modulated by transcription factors which act as a bridge between signaling pathways and gene regulation. Using EnrichR, we compared DEGs from every experimental condition to the transcription factor regulation TRRUST database. We discovered that, in contrast to normoxic conditions, the DEGs under each hypoxic condition are frequently linked to alternative transcription factors, including SP1 and EGR1, in addition to the canonical transcription factors HIF1A and NFKB1 (**Supplementary Figure S4B**). These findings potentially implied that the transcriptome variability of macrophages was enhanced by lower oxygen concentration and longer hypoxia exposure time.

**Figure 5:**
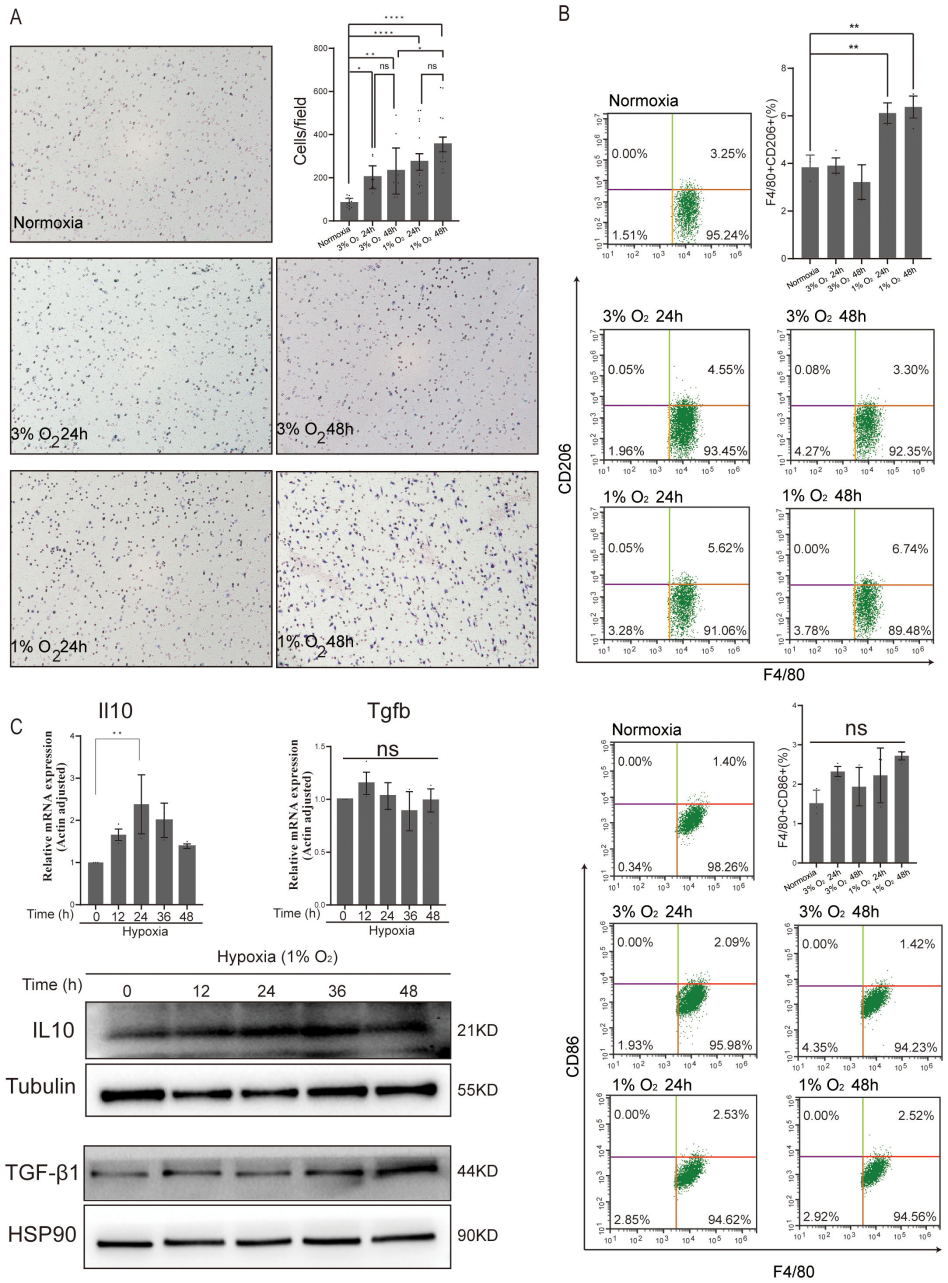
Effect of acute hypoxia on BMDMs 2D migration and polarization. **(A)** Representative images of BMDM migration were captured using microscopy at ×200 magnification, while the number of migratory BMDMs per membrane was quantified under ×400 magnification. Data are from three independent experiments, shown as mean ± SEM. **(B)** Flow cytometry analysis of CD206 (M2 marker) and CD86 (M1 marker) expressed in BMDMs for normoxia, 3% O_2_ 24 h, 3% O_2_ 48 h, 1% O_2_ 24 h, and 1% O_2_ 48 h. Data are from three independent experiments, shown as mean ± SEM. **(C)** The expression levels of anti-inflammatory genes (IL10 and TGF-β1) and the corresponding protein abundance were assessed at various time points under 1% O_2_ exposure. Data are from three independent experiments, shown as mean ± SEM. Significance was determined by one-way ANOVA. *p < 0.05, **p < 0.01, ****p < 0.0001, ns, no signficance.

Further GO and KEGG enrichment analyses were performed on the upregulated and downregulated DEGs in each group, revealing that the upregulated DEGs in macrophages cultured under 1% oxygen for 24 h were found to be primarily enriched in pyruvate metabolic process, cellular response to hypoxia, NADH regeneration process, and HIF-1 signaling and glycolysis metabolic pathway, while the downregulated genes were primarily associated with the process of cell mitosis and cell cycle (**Figure 4B**; **Supplementary Figure S5A**). When the oxygen concentration was adjusted to 3%, the upregulated DEGs remained primarily associated with pyruvate metabolic process, cellular response to hypoxia, and HIF-1 signaling and glycolysis metabolic pathway, while the downregulated genes were mainly related to the extracellular matrix and structure organization (**Figure 4C**; **Supplementary Figure S5B**). As the hypoxic duration extended to 48 h, regardless of whether the oxygen concentration was 1% or 3%, the upregulated DEGs were not only associated with pyruvate metabolic process, cellular response to hypoxia, and HIF-1 signaling but also with chemotaxis, and the downregulated genes at 1% oxygen concentration were linked to cell mitosis and cell cycle process and response to interferon-beta, while those at 3% oxygen concentration were related to response to the peptide (**Figures 4D, E**; **Supplementary Figures S5C, D**). Moreover, the enrichment of upregulated genes in chemotaxis were consistent with the results of the migration assay. Acute hypoxic exposure enhanced the chemotaxis of macrophages, with lower oxygen concentrations leading to more pronounced increases in macrophage chemotaxis (**Figure 5A**).

In comparison to their expression in normoxic macrophages, the analysis of the top 10 most upregulated and downregulated genes under hypoxic conditions showed a significantly higher expression of M2 macrophage marker genes, such as Arg1 (arginase 1) (29), A2m (alpha-2-macroglobulin) (30), and EGLN3 (egl-9 family hypoxia-inducible factor 3) (**Figures 4B–E**) (31). Additionally, compared to normoxic macrophages, hypoxic circumstances also showed a markedly higher expression of the M1 macrophage marker gene Nos2 (nitric oxide synthase 2) (32). To further elucidate the impact of acute hypoxia on macrophage polarization, we analyzed M1 and M2 markers using flow cytometry. The results revealed that the number of M2 macrophages significantly increased only after 24 and 48 h of culture under 1% oxygen. In contrast, the number of M1 macrophages showed no significant increase under acute hypoxia (**Figure 5B**). Analysis of the gene expression of anti-inflammatory factors Il10 and Tgfb in macrophages cultured under 1% oxygen every 12 h revealed that, at the transcriptional level, Il10 expression significantly increased after 24 h of hypoxic exposure, while Tgfb expression showed no significant change. At the protein level, both IL10 and TGF-β1 were significantly upregulated (**Figure 5C**). However, the levels of IL10 or TGF-β1 cytokines in the cell culture supernatant were below the detection limit of ELISA assay kits. These data potentially indicate that acute hypoxia with 1% oxygen could promote the polarization of macrophages toward the M2 phenotype, but the secretion of M2-related cytokines appears to be impaired, potentially due to the absence of secondary stimulation by cytokines such as IL4 or IL13 (33).

### Acute hypoxia macrophages present specific signaling patterns associated with oxygen concentration and exposure time

**Figure 6:**
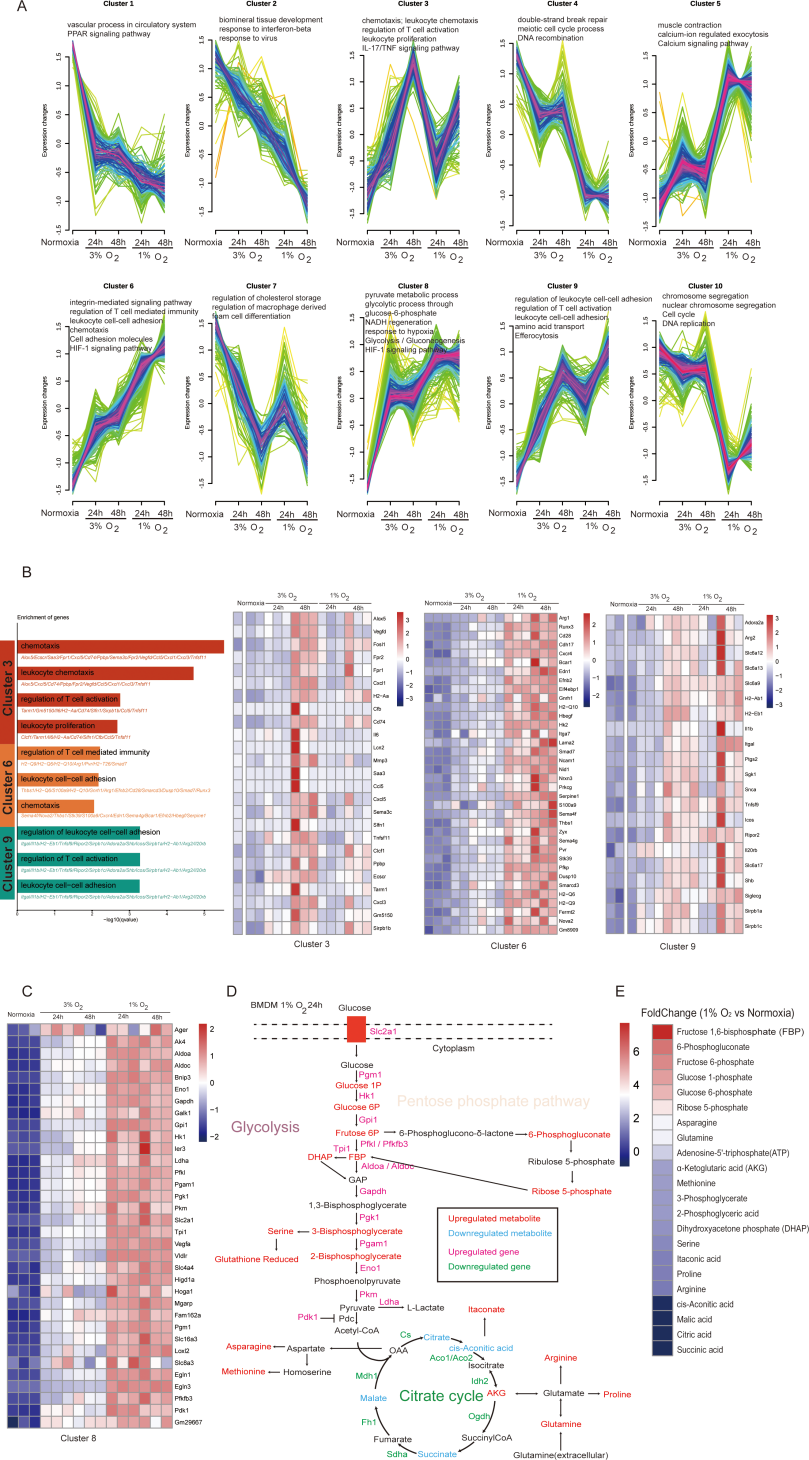
Acute hypoxia leads to metabolic reprogramming of macrophages characterized by enhanced glycolysis and its bypass pathways. **(A)** Fuzzy c-means clustering identified 10 distinct temporal patterns of the differentially expressed genes’ expression. The x-axis represents four hypoxia stages, while the y-axis represents log2-transformed, normalized-intensity ratios in each stage. The top GO (biological process) terms for each cluster are displayed above the charts. **(B)** Genes highly expressed under hypoxic exposure (clusters 3, 6, and 9) are shown in a heatmap. **(C)** The heatmap illustrates the expression patterns of genes associated with metabolic processes within cluster 8. **(D)** Schematic representation of the main metabolic changes of macrophage after 24 h of hypoxic exposure (1% O_2_). **(E)** Fold change of metabolites in macrophages cultured under 1% O_2_ for 24 h compared to those cultured under normoxic conditions.

We noted that the oxygen concentration and exposure duration during acute hypoxia positively correlate with the transcriptome variability of macrophages. We used the fuzzy c-means approach to cluster the DEG expression profiles under all experimental circumstances in order to better analyze the change in signaling status caused by varying oxygen concentrations and exposure durations. Overall, we found 10 unique clusters of specific patterns that correspond to DEGs and exhibit various expression kinetics. Clusters 1, 2, 4, 7, and 10 of these represent downregulated DEGs that are functionally enriched for the PPAR signaling pathway, response to interferon-beta, regulation of cholesterol storage, and meiotic cell cycle process, particularly limiting in the S and G2 phase (**Supplementary Figure S6**). Clusters 5, 6, 8, and 9 represent upregulated DEGs that are enriched for the GO and KEGG terms that describe the calcium signaling pathway, chemotaxis, cell adhesion molecules, HIF-1 signaling pathway, glycolysis and its bypass processes, regulation of T cell activation, and leukocyte cell–cell adhesion. On the other hand, cluster 3 represents DEGs exhibiting a clear uni-modal expression pattern that is enriched for chemotaxis, IL17, and TNF signaling pathway (**Figure 6A**). The results potentially implied that macrophage is highly malleable to oxygen signals and present heterogeneity in immune response. With the exception of those in cluster 3 that are enriched for chemotaxis, IL17, and TNF signaling pathways, which exhibit the opposite direction when macrophages are cultured in 1% oxygen for 24 h, DEGs involved in lipid metabolism, virus defense, and cell division showed a progressive decline during acute hypoxia as the oxygen concentration decreased and the exposure duration increased. In contrast, DEGs enriched in inflammatory response and glucose metabolic process continued to increase (**Figure 6A**).

We enumerated genes in clusters 3, 6, and 9 to further investigate the heterogeneity in immune response of macrophages caused by varying oxygen concentrations and exposure durations during acute hypoxia. We discovered that while DEGs in these clusters enriched for the same items describing biological processes, such as chemotaxis, leukocyte cell–cell adhesion, and regulation of T cell activation, the genes involved between them were clearly different. Cluster 6 primarily consisted of the major histocompatibility complex molecules, such as H2-Q6, H2-Q9, and H2-Q10; cluster 9 primarily consisted of tumor immune microenvironment regulatory genes (Itgal (34), Tnfsf9 (35), Ripor2 (36), and Icos (37)); and cluster 3 primarily consisted of chemokines, such as Cxcl5, Ccl5, Cxcl1, and Cxcl3 (**Figure 6B**). These findings showed that during acute hypoxia, genes related to macrophage immune function show selectivity under varying oxygen concentrations and exposure times.

### Acute-hypoxia-induced metabolic remodeling of macrophage characterized as enhanced glycolysis and its bypass pathways

Macrophages always integrate metabolic and immune signals to perform their diverse roles, and their metabolic state can be a target to modulate immune response in a specific context (38). As anticipated, a frequent feature in macrophages under acute hypoxia is metabolic reprogramming. With an increased tendency accompanied by decreased oxygen concentration and longer exposure duration, DEGs in cluster 8 were enriched for glucose metabolic processes, including glycolysis/gluconeogenesis, glycolytic processes through glucose-6-phosphate, NADH regeneration, and pyruvate metabolic processes (**Figure 6C**). We used LC–MS analyses to identify the metabolites of macrophages exposed to 1% oxygen after 24 h in order to shed light on the specific effects of these DEGs on macrophage metabolism. The findings demonstrated that the transcriptional alterations, which were described as a suppressed tricarboxylic acid cycle and enhanced glycolysis with its bypass pathways, including the pentose-phosphate-pathway and gluconeogenesis-related amino acids, are consistent with the metabolic remodeling that took place in hypoxic macrophages at the transcriptional level (**Figure 6D**). Among the metabolites associated with glycolysis and the pentose phosphate pathway, those exhibiting a fold change greater than 3 after 24 h of culture under 1% oxygen were fructose 1,6-bisphosphate (fold change: 7.8), 6-phosphogluconate (fold change: 6.1), fructose 6-phosphate (fold change: 5.7), glucose 1-phosphate (fold change: 5.3), glucose 6-phosphate (fold change: 4.8), ribose 5-phosphate (fold change: 3.9), asparagine (fold change: 3.3), and glutamine (fold change: 3.2), with metabolites fructose 1,6-bisphosphate, 6-phosphogluconate, and ribose 5-phosphate being key intermediates in the pentose phosphate pathway (**Figure 6E**).

## Discussion

Macrophages are essential for both the innate and adaptive immune responses. They are triggered by the hypoxic signals of wounded or diseased tissues early on and then contribute to the onset and progression of illnesses as well as the healing of injuries. For the development of targeted therapeutics for disorders where low oxygen conditions are prevalent, it is essential to comprehend how acute hypoxia affects the biological behavior of macrophages (39). In order to gain a comprehensive understanding of how macrophages respond to acute hypoxia and how it contributes to the development of disease and injury, we systematically characterized the phenotype of macrophages during acute hypoxia. We discovered that macrophages exposed to acute hypoxia exhibit a variety of phenotypic changes, such as suppressed cell proliferation and phagocytosis function, activated pro- and anti-inflammatory response, transcriptome reprogramming, and metabolic remodeling.

Macrophages exhibit antimicrobial, homeostatic, and immunoregulatory functions, which have significant implications for health and disease (40). Increased proliferation and phagocytosis capacity could contribute to the accumulation of macrophages and exert their biological function efficiently. However, we observed that the cell proliferation and phagocytosis of BMDM were suppressed during acute hypoxia, which is consistent with that in pulmonary alveolar macrophages (41) but contradictory to that in primary peritoneal macrophages (17). The reason for this difference may be the macrophage used in the studies derived from different tissue sources. Furthermore, we discovered that during acute hypoxia, particularly when incubated with 1% oxygen, the genes linked to the mitotic cell cycle in BMDM were downregulated. The cell cycle progression is restricted to the G1–S phase transition, with minimal advancement to G2, according to the flow cytometry results. These findings suggested that BMDM decreases the ability of macrophages to eliminate viruses by inhibiting phagocytosis and cell proliferation, which require more ATP to survive in acute hypoxia. This theory is supported by the fact that the genes responding to interferon-beta and viruses exhibit a dramatic reduction in hypoxia-exposure circumstances at the transcriptional level.

Macrophages are multifunctional cells that support the immune system’s innate and adaptive immune responses (40). To assess the transcriptome-level response to acute hypoxia at varying oxygen concentrations and exposure durations, we used RNA-seq, a systematic approach. The HIF-1 signaling pathway, metabolic remodeling centered on the glycolytic process, and cell chemotaxis under various hypoxia-exposure conditions during acute hypoxia were the most frequently altered signaling pathways. These findings corroborate earlier research that found macrophages to adapt to hypoxia by boosting the glycolysis pathway through HIF-1 to sustain energy production and support cell function (42). We saw an improvement in the pentose phosphate pathway, which is essential for inhibiting oxidative stress via NADPH, using the targeted metabolite identified technique (43). Under 1% oxygen culture, the expression of NADH regeneration genes in macrophages was significantly upregulated, and the levels of intermediate metabolites (fructose 1,6-bisphosphate, 6-phosphogluconate, and ribose 5-phosphate) in the pentose phosphate pathway were markedly increased. This result suggested that the pentose phosphate pathway may be another important metabolic pathway for macrophages to maintain cellular homeostasis during acute hypoxia, particularly under conditions of even lower oxygen concentrations. However, the mechanism by which macrophages utilize the pentose phosphate pathway to maintain cellular homeostasis under acute hypoxia appears to come at the cost of compromised phagocytic function. Recent research by Beielstein et al. has shown that the pentose phosphate pathway inhibition induced increased cell clearance by macrophage phagocytosis (44). Transcription factor enrichment analysis also found that, in addition to the canonical response of macrophages to hypoxia with HIF1A and NFKB1, we also found other transcription factors, such as SP1 and EGR1. SP1 can modulate the expression of genes related to inflammation which has been shown to regulate the microglial/macrophage inflammatory response via the PI3K/AKT/mTOR signaling pathway after intracerebral hemorrhage (45), while EGR1 plays a pivotal role in the function of macrophages, particularly in regulating the inflammatory response and gene expression associated with macrophage activation (46), which need further study to facilitate the understanding of details on the modulatory function of these transcription factors in the immune response of macrophages during acute hypoxia.

We discovered that acute hypoxia could cause the upregulation of both pro- and anti-inflammatory response genes in macrophages. The levels of IL-1β were significantly increased at both transcriptional and translational levels. A recent study has shown that acute suppression of mitochondrial ATP production will provide an essential signal for NLRP3 inflammasome activation to promote the production of IL-1β (47). Here our findings at both transcriptional and metabolic levels indicate that the tricarboxylic acid cycle in macrophages is significantly suppressed under acute hypoxia, potentially leading to an increase in the production of pro-inflammatory factors. To counteract the increased levels of pro-inflammatory factors, macrophages possess corresponding regulatory mechanisms to prevent excessive inflammatory responses. We found that the expression of genes linked to the anti-inflammatory response is significantly higher than that of the pro-inflammatory response, particularly the Arg1 gene, canonical and curated M2 marker gene. This is in line with earlier research showing that hypoxia can encourage the polarization of macrophages to an M2-like phenotype, which is normally linked to anti-inflammation, tissue repair, and regeneration, but in the context of tumors, this can aid in immune evasion and tumor progression (48). A further flow cytometry analysis confirmed that the population of M2 macrophages exhibited a significant increase exclusively after 24 and 48 h of culture under 1% oxygen. The anti-inflammatory cytokines IL10 and TGF-β1, associated with M2 macrophages, showed a significant increase at the protein level, but no detectable levels were observed in the cell culture supernatant. The secretion of cytokines in macrophages is regulated at many levels, including at the level of transcription, translation, and post-translation (49). These results suggested that although acute hypoxia (1% oxygen) induced increased M2 macrophage population, the secretion of M2 macrophage-associated cytokines IL10 and TGF-β1 regulated post-translationally at the endoplasmic reticulum and Golgi and at or near the cell surface may be dampened under hypoxic conditions, warranting further research. In addition, we observed a significant increase in itaconic acid which could inhibit NLRP3 inflammasome to exert anti-inflammatory effects in activated macrophages (50) as an alternate cytokine regulation mechanism of macrophages under acute hypoxia.

Furthermore, we noted that the immune function of macrophages presents three specific signaling patterns associated with hypoxia-exposure conditions in this study. The expression of chemokines such as Cxcl5, Ccl5, Cxcl1, and Cxcl3 present a peak at 3% oxygen for 48 h, which indicated that the immune function of macrophages may be involved in the recruitment of other immune cells in this hypoxic condition (51), while the major histocompatibility complex molecules such as H2-Q6, H2-Q9, and H2-Q10 present a successive increase in hypoxic condition, and the tumor immune microenvironment regulatory genes (Itgal, Tnfsf9, Ripor2, and Icos) showed a slight decrease at 1% oxygen for 24 h. All of these suggested that the immune response of macrophages to acute hypoxia is dependent on the oxygen concentrations and exposure times, which need to be taken into consideration when treating diseases with macrophages as intervention targets in clinical practice.

### Limitations of the study

Although the use of *ex vivo* cells and the absence of *in vivo* functional data limited our study, we used BMDM to systematically illustrate how acute hypoxia affects macrophage proliferation, biological function, transcriptome reprogramming, and metabolic remodeling. This study offers a valuable addition to future *in vivo* research on how macrophages respond to complex tissue microenvironments. The effects of acute hypoxia on RAW264.7’s phagocytosis were inconsistent with those of BMDM despite the fact that both RAW264.7 and BMDM exhibit the same inflammatory response to acute hypoxia. Therefore, the study only used BMDM to examine the effects of acute hypoxia on the transcriptome reprogramming and metabolic remodeling of macrophages.

## Data Availability

The raw sequence data reported in this paper have been deposited in the Genome Sequence Archive (52) in National Genomics Data Center (53), China National Center for Bioinformation/Beijing Institute of Genomics, Chinese Academy of Sciences (GSA: CRA020229) that are publicly accessible at https://ngdc.cncb.ac.cn/.

## Supplementary material

Supplementary Figure 1 Effect of acute hypoxia on the viability and function of RAW 264.7 cells. **(A)** Cell viability was detected in RAW 264.7 cells under normoxic and hypoxic conditions using EdU assay. The proliferative cell nuclei were stained using the EdU assay, with a red stain for proliferative cell nuclei and a blue stain for all nuclei using DAPI (original magnification, ×100). **(B)** RAW 264.7 cells under normoxic and hypoxic conditions were assayed for phagocytosis of fluorescent *Escherichia coli* particles over a 24-h period. Data are from three independent experiments, shown as mean ± SEM. **(C)** ROS production levels and NAD+/NADH ratio were detected in RAW 264.7 cells under normoxic and hypoxic conditions. Data are from three independent experiments, shown as mean ± SEM. Significance was determined by one-way ANOVA in **(A–C)**. **p* < 0.05, ***p* < 0.01.

Supplementary Figure 2 Lactate dehydrogenase (LDH) activity of BMDMs at various time points under 1% O_2_ exposure. Data are from three independent experiments, shown as mean ± SEM. Significance was determined by one-way ANOVA. **p* < 0.05, *****p* < 0.0001.

Supplementary Figure 3 Validation of the differentially expressed genes identified by RNA-seq data under normoxic and 1% O_2_ conditions **(A)** and 3% O_2_ conditions **(B)** using RT-qPCR.

Supplementary Figure 4 GO (biological process) and transcription factors enrichment analysis for specific differentially expressed genes in oxygen concentrations and exposure times. **(A)** GO (biological process) enrichment analysis. **(B)** Transcription factors enrichment analysis. Enrichment generated with EnrichR and drawn from the TRRUST database of transcription factors.

Supplementary Figure 5 KEGG pathway enrichment analysis for differentially expressed genes in the comparison of 1% O_2_ 24 h vs. normoxia **(A)**, 3% O_2_ 24 h vs. normoxia **(B)**, 1% O_2_ 48 h vs. normoxia **(C)**, and 3% O_2_ 48 h vs. normoxia **(D)**.

Supplementary Figure 6 Acute hypoxia suppressed the cell cycle of macrophage. The cell cycle was detected by flow cytomery. Data are from three independent experiments, shown as mean ± SEM. Significance was determined by one-way ANOVA in **(A–D)**. ***p* < 0.01, ****p* < 0.001.
