# Supplementary material for: Acute hypoxia modulate macrophage phenotype accompanied with transcriptome re-programming and metabolic re-modeling
Source: Front Immunol. 2025 Feb 17;16:1534009. doi: 10.3389/fimmu.2025.1534009 (PMC11872928; doi:10.3389/fimmu.2025.1534009)
Supplement: Supplementary file 1 [file Table1.docx]

| **Target** | **Source** | **Forward(5’ to 3’)** | **Reverse(5’ to 3’)** |
| --- | --- | --- | --- |
| Mouse:Cox2 | Primer 5.0 | TGCACTATGGTTACAAAAGCTGG | TCAGGAAGCTCCTTATTTCCCTT |
| Mouse:Nos2 | Primer 5.0 | GAGCCACAGTCCTCTTTGCT | CAACCTTGGTGTTGAAGGCG |
| Mouse:IL6 | Primer 5.0 | CCAAGAGGTGAGTGCTTCCC | CTGTTGTTCAGACTCTCTCCCT |
| Mouse:IL1β | Primer 5.0 | GCAACTGTTCCTGAACTCAACT | ATCTTTTGGGGTCCGTCAACT |
| Mouse:Arg1 | Primer 5.0 | CTCCAAGCCCAAAGTCCTTAGAG | AGGAGCTGTCATTAGGGACATC |
| Mouse:Gipr | Primer 5.0 | AAAGATGTTGGAGACCACAGAAC | GCAGACACCTGACGGAACC |
| Mouse:H2-Q10 | Primer 5.0 | ACGTGGCGGCGATTATCAC | AGGTAGGCCCTGTAATACTCTG |
| Mouse:H2-Q2 | Primer 5.0 | GAAGCCTACTCCGAGGGTATG | CGTCAAGCAATCGACCTCCA |
| Mouse:Ache | Primer 5.0 | CTCCCTGGTATCCCCTGCATA | GGATGCCCAGAAAAGCTGAGA |
| Mouse:A2m | Primer 5.0 | GAGATGCAGCTTGATGTGAATGC | CACCTGCCCAACGAAAGGAA |
| Mouse:Milp | Primer 5.0 | CTGCCCGACCTTCTCCCTT | TCTGCTCTGTAGACCCCGTTG |
| Mouse:Ackr4 | Primer 5.0 | TCCTGCCTGCCTTCTTCACA | TCGGTCTTGGTCCTCTGCTTC |
| Mouse:Adipoq | Primer 5.0 | TGTTCCTCTTAATCCTGCCCA | CCAACCTGCACAAGTTCCCTT |
| Mouse:Enpep | Primer 5.0 | ATAGTGGGACTTTCTGTGGGT | GGTCGTAGTGAACTGGATTGATG |
| Mouse:Abi3bp | Primer 5.0 | AGTTCCTGCGTCCAAATGCAA | CGACTATCAGATACTTCGGCTCT |
| Mouse:Plin1 | Primer 5.0 | CTGTGTGCAATGCCTATGAGA | CTGGAGGGTATTGAAGAGCCG |
| Mouse:Il10 | Primer 5.0 | GCTCTTACTGACTGGCATGAG | CGCAGETCTAGGAGCATGTG |
| Mouse:Tgfb | Primer 5.0 | CTCCCGTGGCTTCTAGTGC | GCCTTAGTTTGGACAGGATCTG |

Supplementary Table 1:Primers for quantitative RT-PCR
