# Supplementary figures and images for: Acute hypoxia modulate macrophage phenotype accompanied with transcriptome re-programming and metabolic re-modeling
Source: Front Immunol. 2025 Feb 17;16:1534009. doi: 10.3389/fimmu.2025.1534009 (PMC11872928; doi:10.3389/fimmu.2025.1534009)

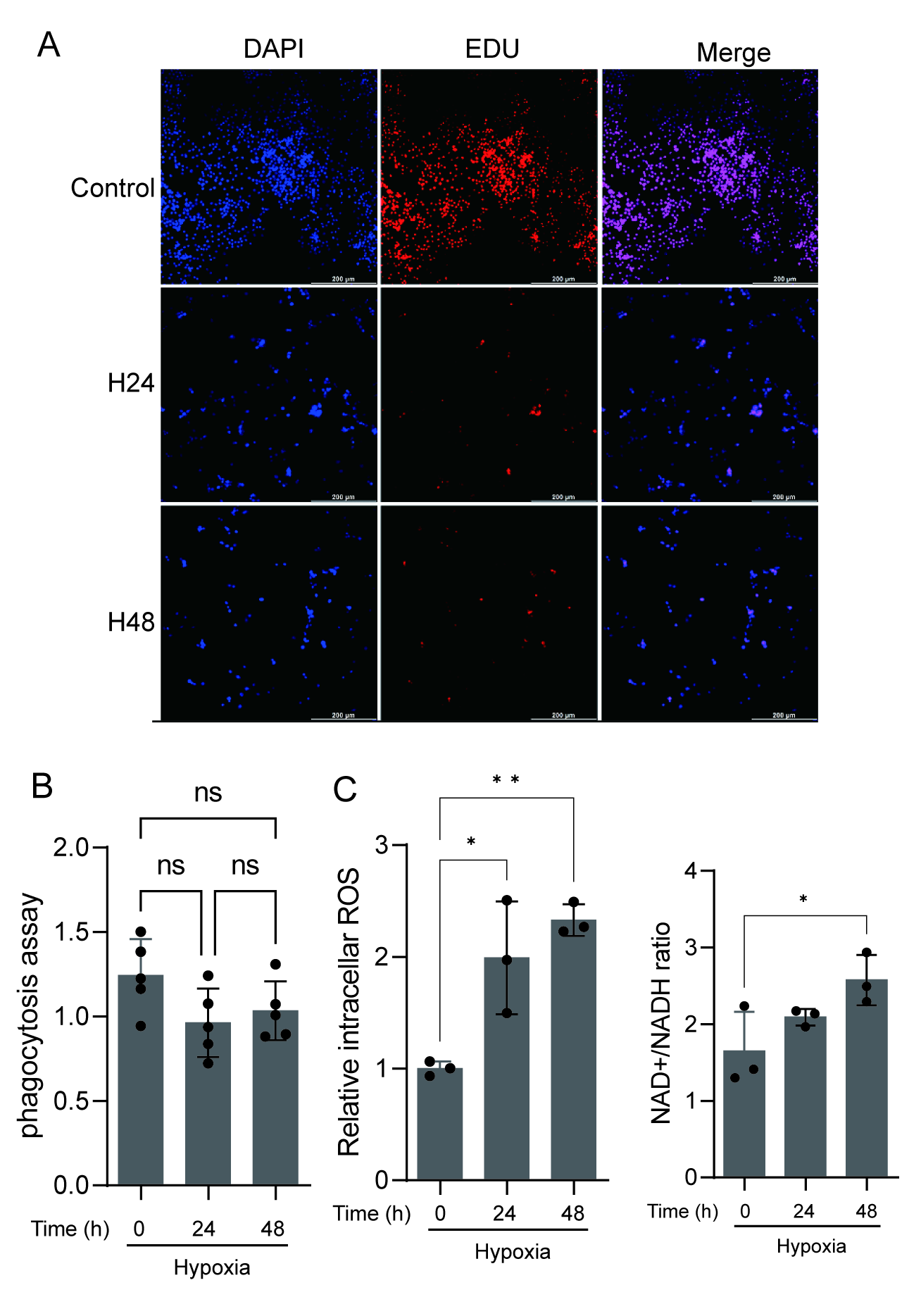

Supplement: Supplementary Figure 1 — Effect of acute hypoxia on the viability and function of RAW 264.7 cells. (A) Cell viability was detected in RAW 264.7 cells under normoxic and hypoxic conditions using EdU assay. The proliferative cell nuclei were stained using the EdU assay, with a red stain for proliferative cell nuclei and a blue stain for all nuclei using DAPI (original magnification, ×100). (B) RAW 264.7 cells under normoxic and hypoxic conditions were assayed for phagocytosis of fluorescent Escherichia coli particles over a 24-h period. Data are from three independent experiments, shown as mean ± SEM. (C) ROS production levels and NAD+/NADH ratio were detected in RAW 264.7 cells under normoxic and hypoxic conditions. Data are from three independent experiments, shown as mean ± SEM. Significance was determined by one-way ANOVA in (A–C). *p < 0.05, **p < 0.01. [file Image1.tif]

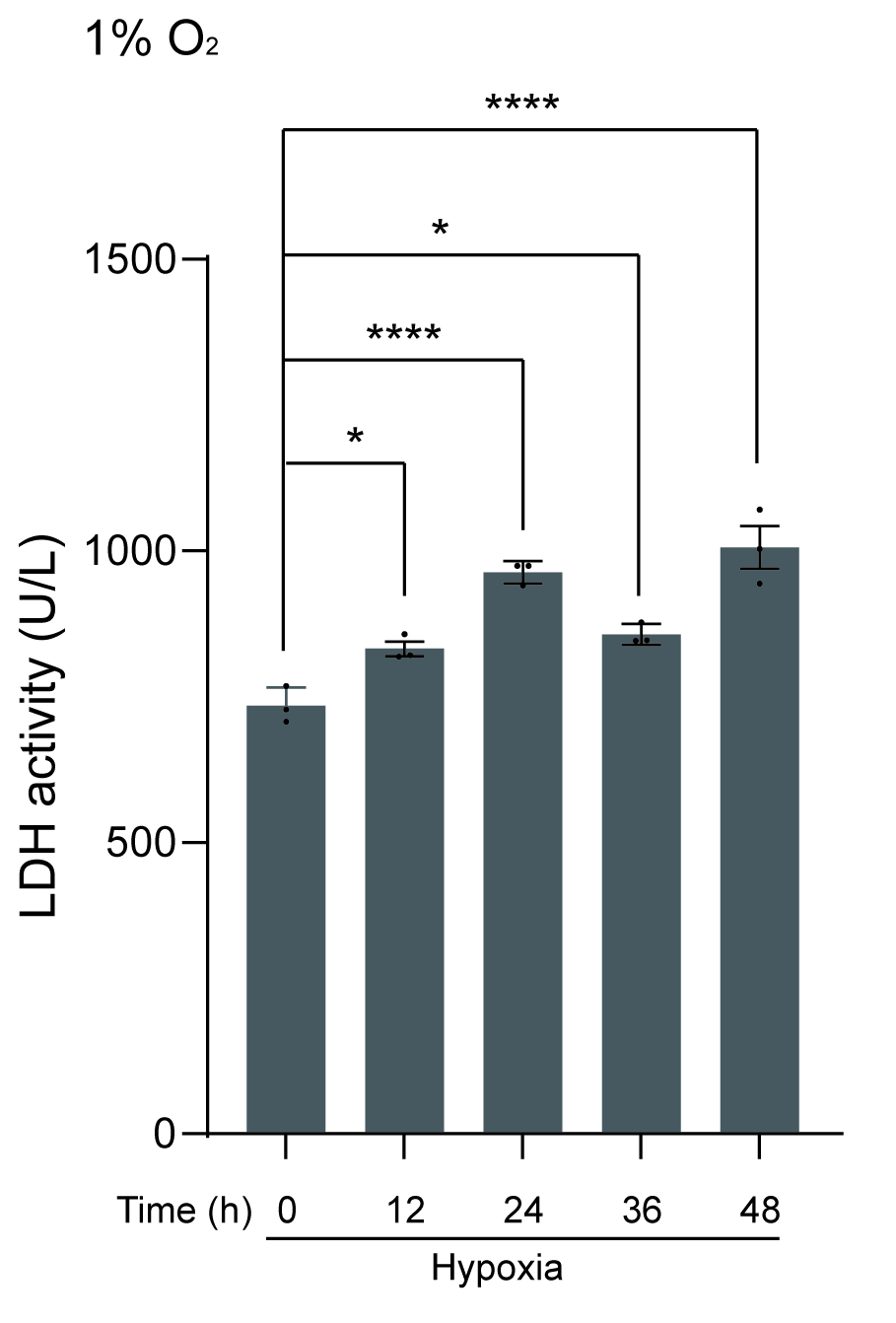

Supplement: Supplementary Figure 2 — Lactate dehydrogenase (LDH) activity of BMDMs at various time points under 1% O2 exposure. Data are from three independent experiments, shown as mean ± SEM. Significance was determined by one-way ANOVA. *p < 0.05, ****p < 0.0001. [file Image2.tif]

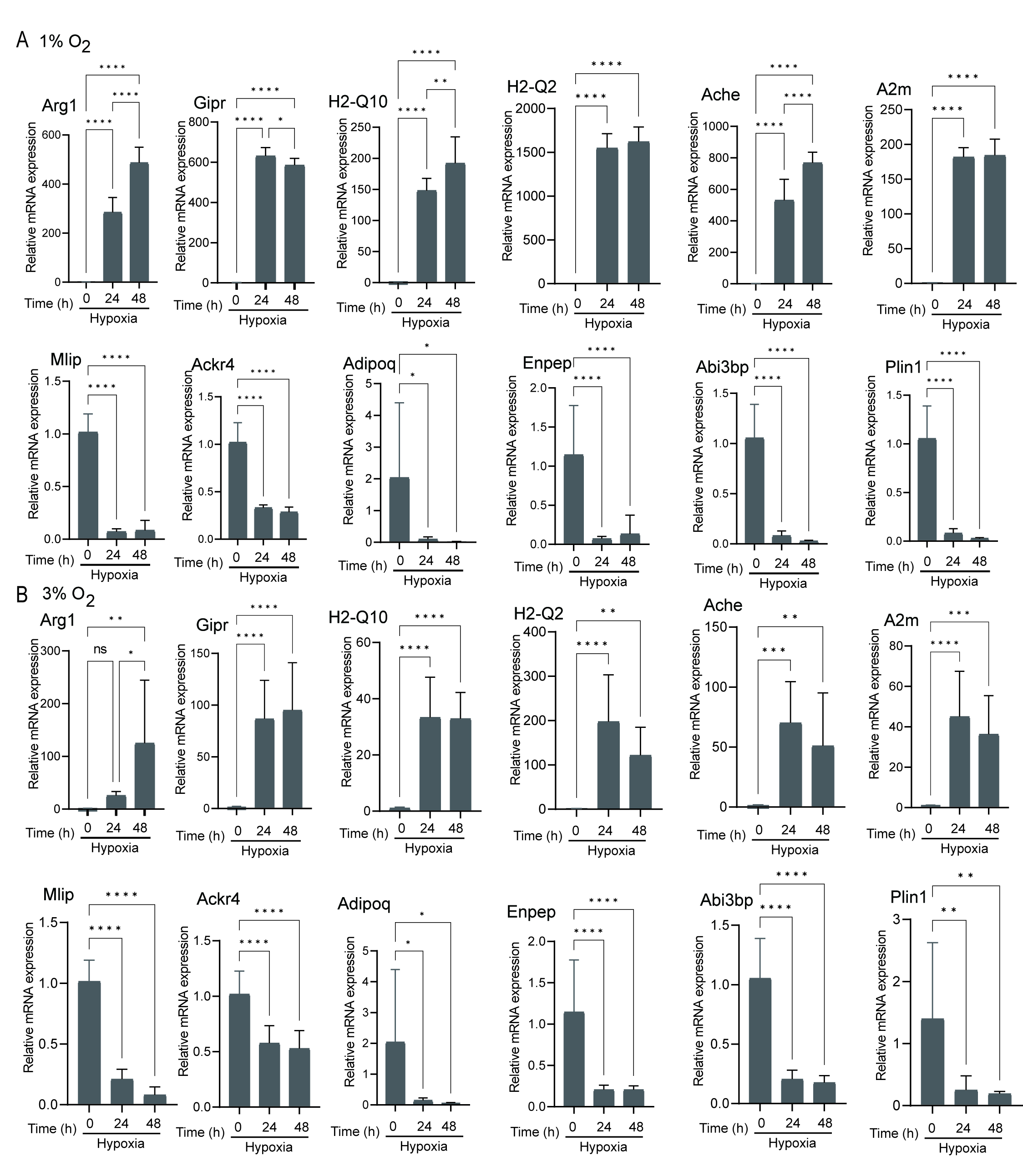

Supplement: Supplementary Figure 3 — Validation of the differentially expressed genes identified by RNA-seq data under normoxic and 1% O2 conditions (A) and 3% O2 conditions (B) using RT-qPCR. [file Image3.tif]

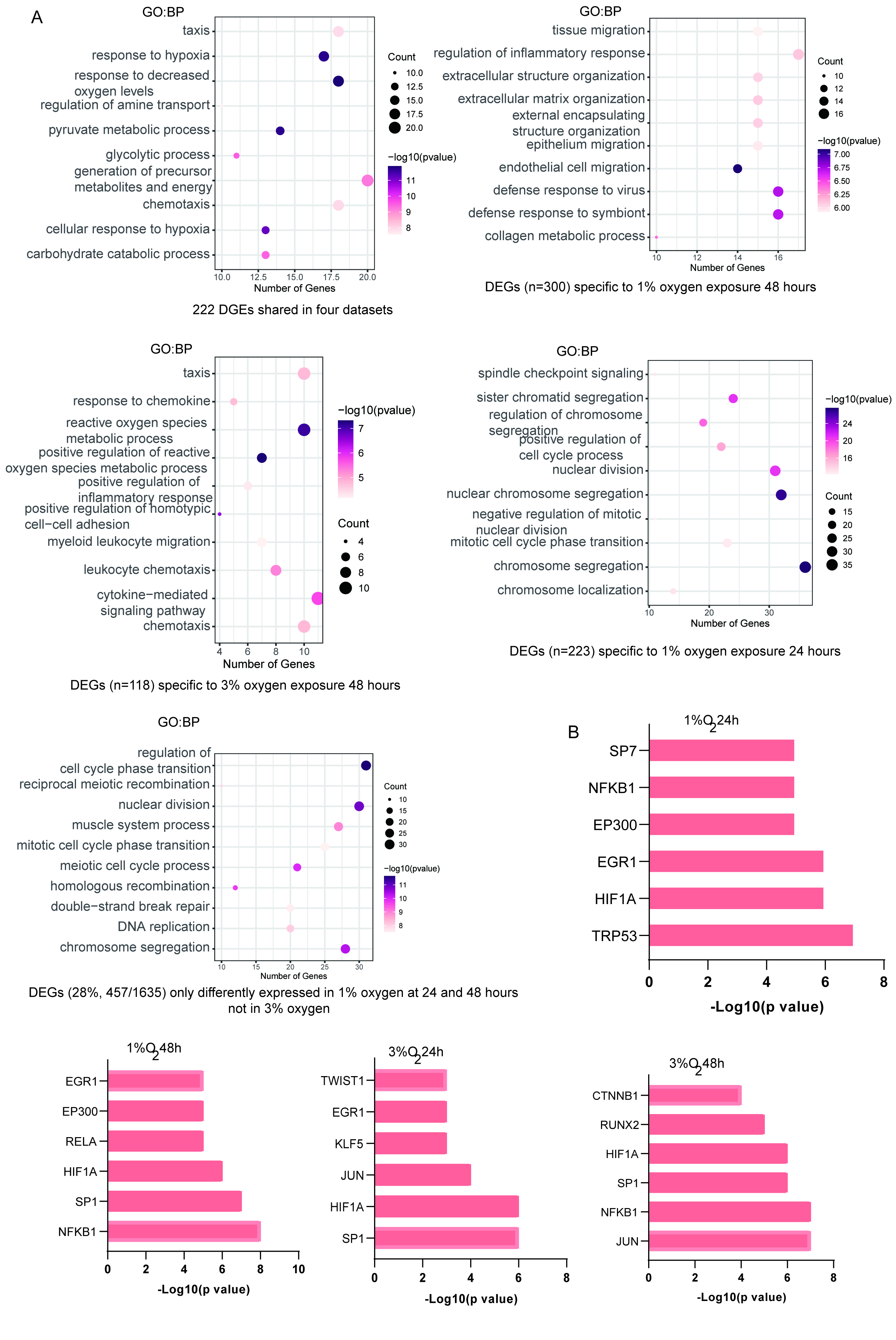

Supplement: Supplementary Figure 4 — GO (biological process) and transcription factors enrichment analysis for specific differentially expressed genes in oxygen concentrations and exposure times. (A) GO (biological process) enrichment analysis. (B) Transcription factors enrichment analysis. Enrichment generated with EnrichR and drawn from the TRRUST database of transcription factors. [file Image4.tif]

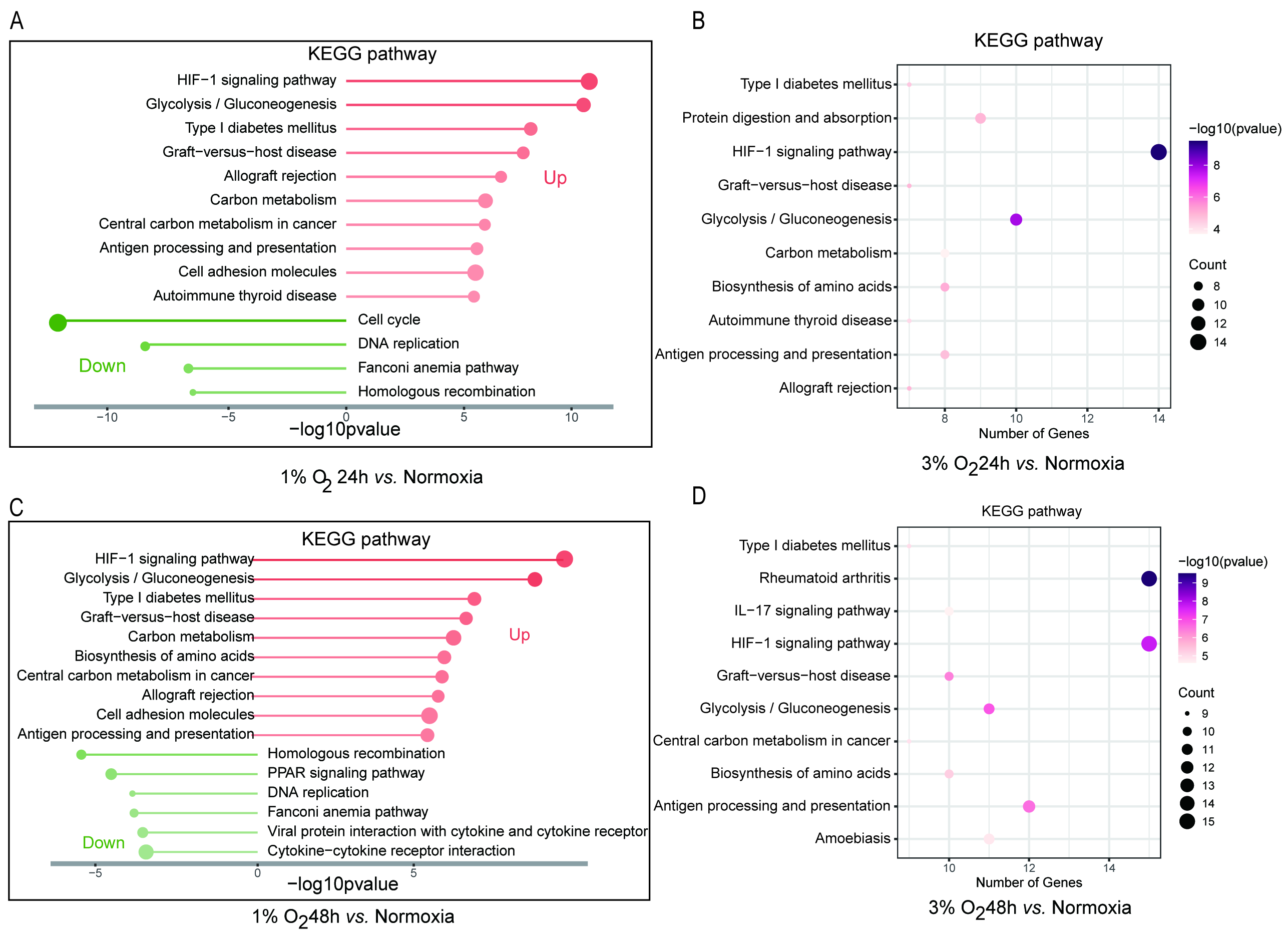

Supplement: Supplementary Figure 5 — KEGG pathway enrichment analysis for differentially expressed genes in the comparison of 1% O2 24 h vs. normoxia (A), 3% O2 24 h vs. normoxia (B), 1% O2 48 h vs. normoxia (C), and 3% O2 48 h vs. normoxia (D). [file Image5.tif]

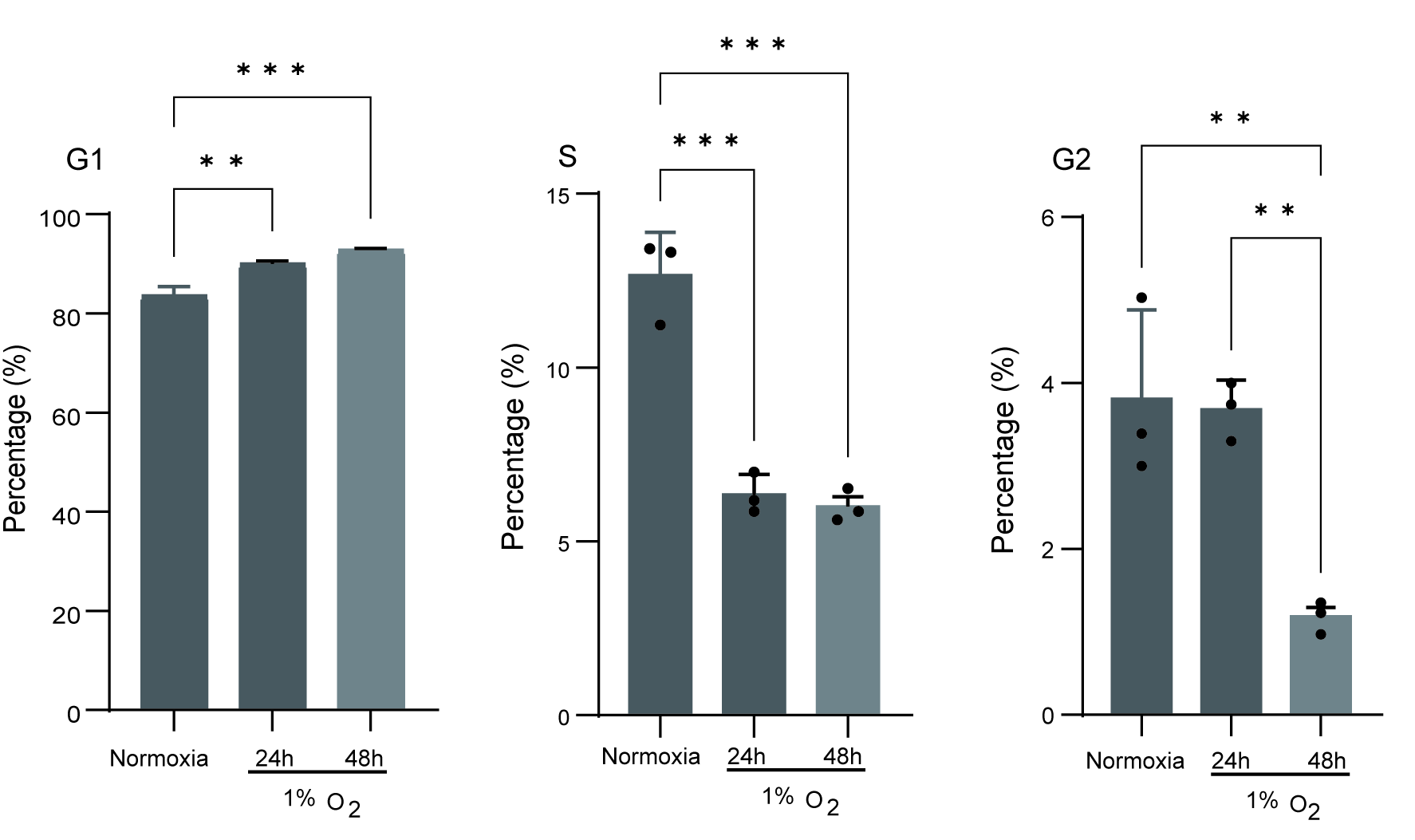

Supplement: Supplementary Figure 6 — Acute hypoxia suppressed the cell cycle of macrophage. The cell cycle was detected by flow cytomery. Data are from three independent experiments, shown as mean ± SEM. Significance was determined by one-way ANOVA in (A–D). **p < 0.01, ***p < 0.001. [file Image6.tif]
